# Associations of perceived stress with depression in medical students: the mediating role of rumination and the moderating role of emotional intelligence

**DOI:** 10.3389/fpsyg.2025.1620067

**Published:** 2025-07-31

**Authors:** Youjuan Hong, Lina Luo, Zixuan Li, Siyu Wu, Xiaofan Bao

**Affiliations:** ^1^School of Nursing, Fujian Medical University, Fuzhou, China; ^2^Research Center for Nursing Humanity, Fujian Medical University, Fuzhou, China

**Keywords:** perceived stress, rumination, emotional intelligence, depression, medical students

## Abstract

**Background:**

Medical students have become a group with a high prevalence of depression and are particularly vulnerable to it. Recognizing the factors affecting depression among medical students is crucial. This study was aimed at exploring the effects of perceived stress on medical students’ depression under the mediating role of the rumination and the moderating role of the emotional intelligence.

**Methods:**

A survey was conducted with 648 medical students in XX Province, XX (blind review). Participants provided responses to measures of perceived stress, rumination, emotional intelligence, and depression. Data analysis was performed in SPSS version 26 and the SPSS PROCESS Macro.

**Results:**

The results revealed significant positive associations between perceived stress (*r* = 0.63, *p* < 0.01) and rumination (*r* = 0.59, *p* < 0.01) with depression. Rumination plays a mediating role between perceived stress and depression, with the mediation effect accounting for 31.67% (SE = 0.10, 95% CI = 0.15, 0.26). Furthermore, emotional intelligence significantly moderated the direct effect (moderated mediation = −0.01, SE = 0.01, 95% CI = −0.01, −0.00).

**Conclusion:**

Rumination serves as a mediator in the relationship between perceived stress and depression, while emotional intelligence significantly moderates the impact of perceived stress on depression.

## 1 Introduction

Depression represents a prevalent mental health concern among college students. Existing literature indicates a higher prevalence of depression among medical students compared to the general population (Tam et al., 2019; Rotenstein et al., 2016). The prevalence of depression among medical students in China exceeds 20%, with a consistent upward trend over the last decade (Gao et al., 2020; Chen et al., 2022). Depression among medical students poses a significant risk, potentially affecting their health, academic performance, and identity formation, professional identity, a heightened tendency toward cynicism and decreased empathy in doctor-patient interactions (Pitanupong et al., 2024; Lo Moro et al., 2022) and the effectiveness of future healthcare services (Liu et al., 2021). The mental health of medical students deserves increased focus and care as they prepare to join the healthcare workforce (Chen et al., 2022; Liu et al., 2021). Previous research found that perceived stress are associated with an increased risk of experiencing negative emotional states, ultimately contributing to depressive symptoms in college students (Liu et al., 2021). Individuals’ cognitive interpretation and perception of a stressful event shape the impact of objective stressors on them (Cohen et al., 1983; Wang et al., 2021). However, Previous studies have primarily focused on the effects of stress-related life events and stress sources on depression among medical students (de Sa e Camargo et al., 2023), with relatively less exploration of its impact on depression from the perspective of perceived stress (Li et al., 2023; Elayoubi et al., 2023; Schwaab et al., 2022). Therefore, our study employed mediation and moderation analyses to investigate the interrelationships among perceived stress, rumination, emotional intelligence, and depression in medical students. This research aims to deepen our understanding of depression in this population, offering insights for the prevention of future health problems, stress reduction, and the improvement of emotional regulation skills, all of which are essential for enhancing academic performance.

Depression refers to a common emotional experience characterized by persistent feelings of sadness, despair, and hopelessness (Mirza et al., 2021). Psychological perspectives of stress argued that, regardless of the stressful situation, the response to stress largely depends upon how that situation is perceived. Perceived stress is the extent to which an individual evaluates an external event as a source of stress (Cohen et al., 1983). Perceived stress offers valuable insights beyond objective indices of exposure to stress-inducing events, as it encapsulates an individual’s subjective appraisal of these pressures and their expected ability to effectively navigate these challenges (Hu et al., 2014). Studies have indicated that an increase in perceived stress levels is linked to a higher prevalence of depression (Cristóbal-Narváez et al., 2020). Prior research has consistently shown that medical students encounter heightened psychological stress in comparison to their counterparts in other academic disciplines and the general population across various countries worldwide (Azim and Baig, 2019; Gazzaz et al., 2018). Particularly, medical students often face heightened levels of perceived stress due to the academic demands, limited free time, vast study materials, and frequent exams in a highly competitive environment (Steiner-Hofbauer and Holzinger, 2020; Bali et al., 2020; Iqbal et al., 2015). Medical students may experience higher levels of depressive symptoms due to increased levels of perceived pressure.

Rumination refers to the repetitive contemplation of one’s negative mood, along with the exploration of potential origins and repercussions of distress (Nolen-Hoeksema et al., 2008). In particular, people with high levels of rumination often passively dwell on their negative emotions and the accompanying symptoms of distress (e.g., “I am worthless and feel hopeless”). Moreover, they experience heightened upset as they seek meaning in their distress (e.g., “When will this misery end?”). This is in accordance with the perseverative cognition hypothesis, suggesting that iterative cognitive patterns, such as rumination, could contribute to mental health challenges (e.g., heightened depressive mood and pessimism) and susceptibility to illness by intensifying immediate reactions, impeding recovery, or reinstating responses following exposure to a stressor (Clancy et al., 2016; Modini et al., 2018; McLaughlin et al., 2007). As a result, the melancholic feelings experienced by individuals with depression are heightened when they engage in ruminative thought patterns, contributing to prolonged episodes and the development of severe depression. In particular, those with a greater inclination towards rumination are more prone to entering a harmful cycle of negative emotions when faced with stressful events, possibly leading to the onset of depression (Nolen-Hoeksema et al., 2008). The relationship between stress and depression may be mediated by rumination, which can reactivate or prolong psychological responses to stress, and these, in turn, are associated with the development of depression (Berset et al., 2011).

Diathesis-stress models are frequently depicted as comprehensive developmental frameworks, suggesting that various risk factors throughout the developmental process interact with stressors and protective elements. These interactions contribute to either typical development or the emergence of psychopathological conditions (Kendler, 2020). Individual traits (e.g., emotional intelligence) may moderate the relationship between perceived stress and depression (Joormann and Stanton, 2016; Strauman and Eddington, 2017). Emotional intelligence involves the capacity to accurately recognize, differentiate, and understand personal and other people’s emotions, using this awareness to guide cognitive processes and behavioral responses (Salovey and Mayer, 1989-1990). Emotional intelligence plays a role in reducing anxiety and facilitating recovery from depressive episodes and mood fluctuations (Estévez et al., 2018; Gomez-Baya et al., 2017). It exhibits a negative association with depression and has been identified as a significant predictor of depression in adolescents (Abdollahi et al., 2015; Afolabi and Balogun, 2017). Individuals with high emotional intelligence can effectively buffer the negative impact of perceived stress on depressive symptoms through enhanced emotional awareness, emotional understanding, and adaptive emotion regulation strategies (e.g., cognitive reappraisal). Conversely, individuals with low emotional intelligence, due to their deficiency in effectively identifying, understanding, and managing their own and others’ emotions, often struggle to adopt adaptive coping strategies when facing stress, consequently making them more susceptible to depressive states. It has been indicated by previous studies that emotional intelligence acts as a moderator in the connection between stress and mental health.

**Figure 1:**
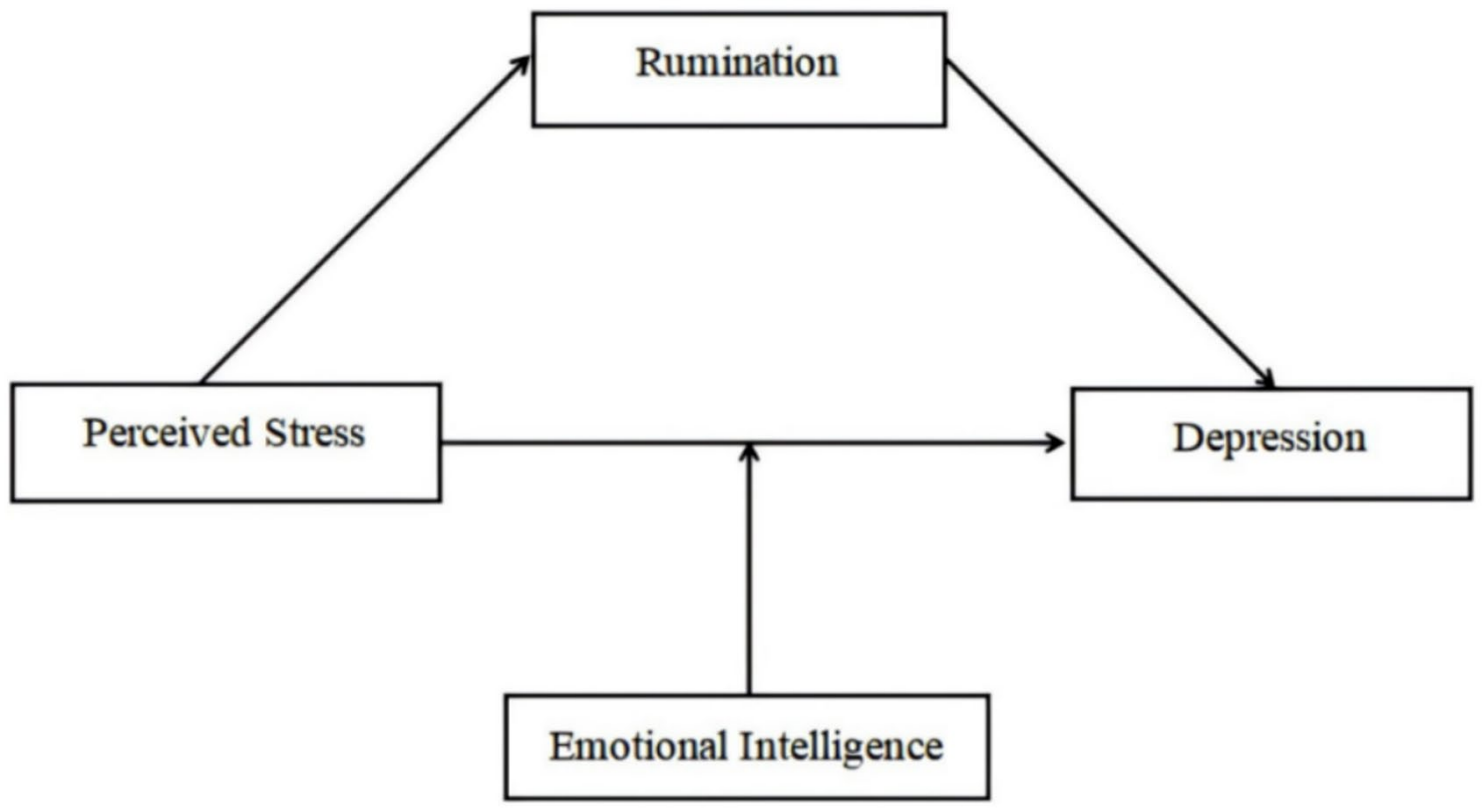
Hypothesized research model.

To our knowledge, no research has examined the inner mechanism between perceived stress and depression in medical students. Expanding upon existing literature, we present a moderated mediation model, which posits that the effect of perceived stress on depression, mediated by rumination, may differ according to the level of emotional intelligence (see Figure 1). The following three hypotheses are proposed by the study:

*Hypothesis 1*: Perceived stress was an important positive predictor of depression among medical students.

*Hypothesis 2*: Rumination acted as an intermediary in the correlation between perceived stress and depression in medical students.

*Hypothesis 3*: Emotional intelligence acted as a buffer in the connection between perceived stress and depression among medical students.

## 2 Methods

### 2.1 Participants and procedure

The sample comprised 648 medical students, of whom 473 (73.00%) were female and 175 (27.00%) were male. The participants’ average age was 19.76 years (SD = 1.73), and their ages varied between 18 and 27 years. Regarding academic year, 202 participants (31.17%) were first-year students, 173 (26.70%) were second-year students, 178 (27.47%) were third-year students, and 95 (14.66%) were fourth-year students. Additionally, 334 participants (48.46%) resided in urban areas, while 314 (51.54%) were from rural areas.

Data were collected from February 2024 to October 2024. Participants included 648 medical students from freshman to senior year in three medical universities. The researchers initiated contact with the participants and conducted explanatory sessions to elucidate the study’s purpose and significance. Subsequently, self-reporting questionnaires assessing perceived stress, rumination, emotional intelligence, and depression were disseminated among the medical students. Demographic details encompassing gender, age, and academic grade were gathered. Participants were informed that their responses would be kept confidential and that they had the right to discontinue participation in the study at any time. After fully understanding the study’s objectives, participants gave their written informed consent. The questionnaires were completed within a 15-min period, and the researchers collected the completed responses.

The study was approved by the Academic Ethics Committee of Fujian Medical University. Upon completing the survey, participants were given a small token gift as a gesture of appreciation. In total, 660 students agreed to participate and completed the questionnaires, resulting in a response rate of 98.18%, with 12 invalid questionnaires (containing more than six missing items) excluded from the final analysis.

### 2.2 Measurements

#### 2.2.1 Perceived stress

The Perceived Stress Scale is a well-established tool for assessing perceived stress, initially developed by Cohen et al. (1983). The scale consists of 14 items, each of which is assessed on a 5-point Likert scale, with 0 representing never and 4 representing often. The cumulative score, ranging from 0 to 56, is computed by summing the scores of all individual items. Higher scores on the scale reflect increased degrees of perceived stress (Liu et al., 2021; Meng et al., 2020). In this study, an assessment of internal consistency resulted in a Cronbach’s alpha coefficient of 0.83, affirming robust internal reliability.

#### 2.2.2 Rumination

Rumination was assessed using the Chinese version of the Ruminative Responses Scale (Nolen-Hoeksema and Morrow, 1991; Han and Yang, 2009), which comprises three dimensions of rumination (symptom rumination, brooding, and reflective pondering) and 22 items. Each item was scored on a 4-point Likert scale (1 for never, 4 for always). The higher the participant’s score, the higher the tendency to ruminate cognitive style was reflected. In this study, the scale demonstrated a Cronbach’s alpha of 0.89.

#### 2.2.3 Emotional intelligence

Emotional intelligence was assessed using the Chinese version of the Emotional Intelligence Scale (Schutte et al., 2007), which includes 33 self-report items assessing emotional perception, the management of one’s own and others’ emotions, and utilization of one’s emotions. Each item was evaluated on a 5-point scale spanning from 1 (strongly disagree) to 5 (strongly agree). Example items consist of, “I can control my emotions” and “I am typically very clear about my feelings.” Higher scores on this scale indicate greater emotional intelligence. In this study, the scale demonstrated a Cronbach’s alpha of 0.86.

#### 2.2.4 Depressive

The Chinese version of Self-Rating Depression Scale (SDS) (Zung, 1965) was used to assess depressive symptoms. The scale comprises 20 questions which is scored using a 4-point Likert scale (1 = none or very little, 4 = most or all of the time). Ten items are worded in a positive manner, while the other ten are worded negatively. The scores for the positively worded items are reversed, yielding total scores ranging from 20 to 80, with more severe depressive symptoms being indicated by higher scores. In the current study, the scales’ Cronbach’s alpha was 0.84.

### 2.3 Statistical analysis

Correlation analysis was performed with SPSS 26.0, while the PROCESS Macro v2.16.3 Model 4 and Model 5 were used to test the moderated mediation model, utilizing 5,000 bootstrap samples (Hayes, 2013).

## 3 Results

### 3.1 Common method bias test

As all the data collected in this research were based on self-reported measures, the potential for bias due to measurement method was evaluated through the application of Harman’s single-factor test (Chang et al., 2010; Podsakoff et al., 2003). It was indicated by the first unrotated factor, which explained 18.58% (<40%) of the variance. The result showed that there was not a considerable risk to the validity of the study.

### 3.2 Descriptive statistics and correlations

As presented in Table 1, perceived stress significantly positively correlated with depression (*r* = 0.64, *p* < 0.001). Consequently, H1 received support.

**Table 1:** Descriptive statistics.

Variables	*M*	SD	1	2	3	4
1. Perceived stress	2.79	0.53	1			
2. Rumination	2.06	0.48	0.50^***^	1		
3. Emotional intelligence	3.67	0.44	−0.35^***^	−0.24^***^	1	
4. Depression	2.32	0.50	0.64^***^	0.57^***^	−0.52^***^	1

M, mean; SD, standard deviation. ^***^ *p* < 0.001.

### 3.3 The mediation of rumination

Prior to analysis, all measures were standardized, with control implemented for three demographic factors (gender, age and year of class). The result showed that year of class was positively correlated with depression and rumination. Perceived stress significantly positively predicts rumination (*β* = 0.46, *p* < 0.001). When rumination is introduced as a mediating variable, the predictive effect of perceived stress on depression remains significant (*β* = 0.41, *p* < 0.01), suggesting that rumination partially mediates the relationship between perceived stress and depression. To further assess the significance of rumination’s mediating effect, the bias-corrected percentile bootstrap method (with 5,000 repetitions) was used, revealing a mediating effect of 0.17 with a 95% confidence interval of [0.13, 0.23]. The fact that 0 is not included in the confidence interval indicates that the relationship between perceived stress and depression is significantly mediated by rumination. These results suggest that perceived stress directly influences depression and also exerts an indirect effect through rumination. The immediate effect magnitude is 0.41, accounting for 70.69% of the total effect size (0.58), while the mediating effect size is 0.17, contributing 29.31% of the overall impact. Thus, H2 is supported (see Table 2).

**Table 2:** Examining the mediating effect.

Predictors	Dependent variable (depression)		Mediator variable (rumination)		Dependent variable (depression)	
	*β*	*t*	*β*	*t*	*β*	*t*
Gender	0.15	1.07	−0.10	−2.75	0.04	1.38
Age	0.01	0.56	−0.01	−0.21	−0.01	−0.99
Year of class	0.87	2.81*	0.03	0.06	0.80	2.29*
Perceived stress	0.61***	20.66	0.46***	14.29	0.41***	12.29
Rumination					0.38***	10.12
*R* ^2^	0.41		0.27		0.50	
*F*	148.57***		52.35***		111.61***	

*p < 0.05, ***p < 0.001.

### 3.4 The moderation of emotional intelligence

**Figure 2:**
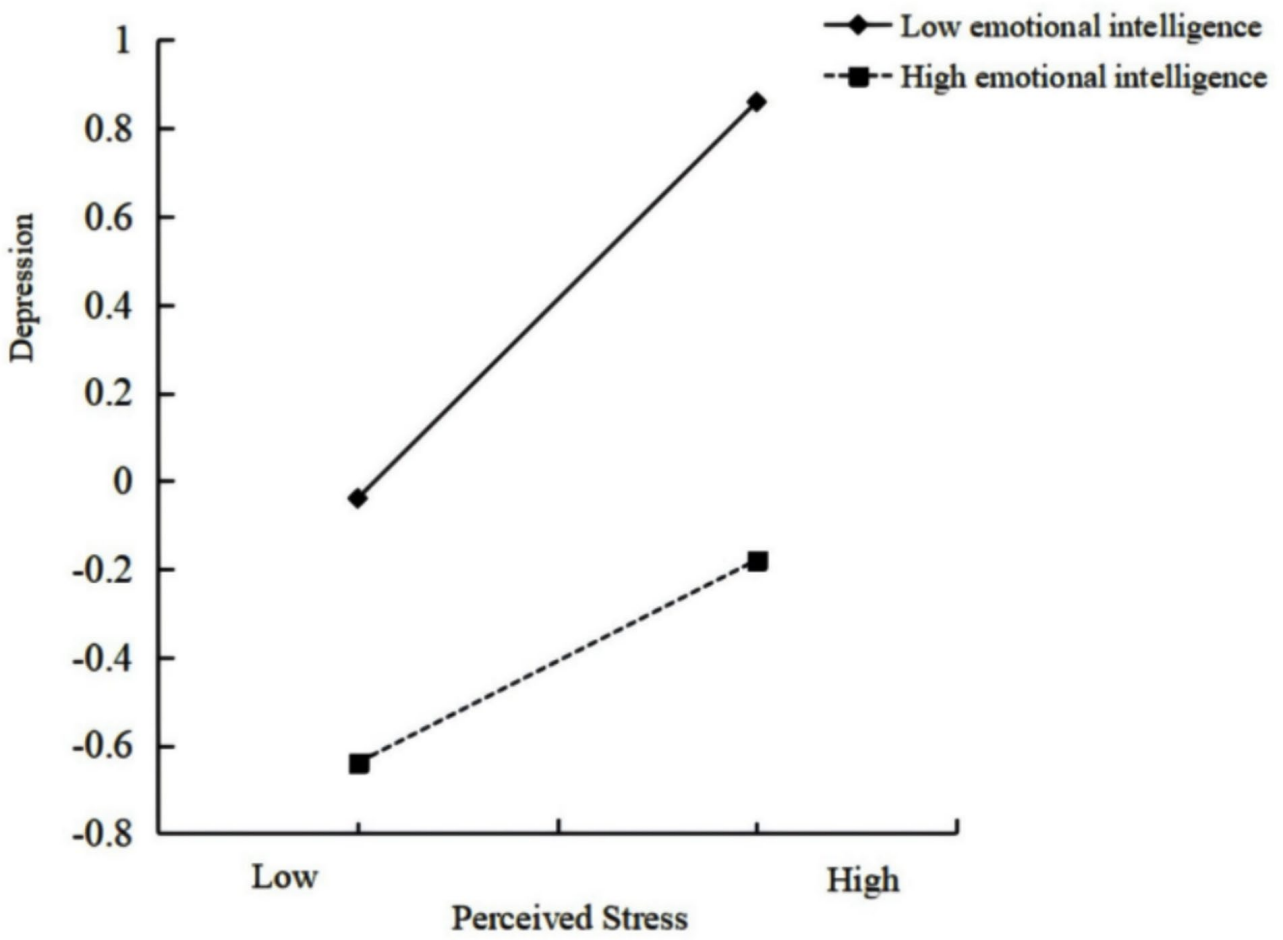
Interaction between perceived stress emotional intelligence.

Demographic characteristics including gender, age, and year of class were included as covariates in the preliminary analyses. Results indicated that higher grade levels were significantly associated with elevated depression. Perceived stress demonstrated a positive influence in predicting depression (*β* = 0.30, *p* < 0.05), while emotional intelligence exhibited a negative impact on forecasting depression (*β* = −0.41, *p* < 0.05) (see Table 3). Additionally, the interaction effect between perceived stress and emotional intelligence on depression reached statistical significance (*β* = −0.11, *p* < 0.001). This outcome suggests that emotional intelligence moderated the direct influence of perceived stress on depression. Consequently, H3 garnered support. Moreover, a simple slope analysis was employed to scrutinize the moderating influence of emotional intelligence. A score greater than the mean plus one standard deviation (*M* + SD) was classified as the high group, while a score lower than the mean plus one standard deviation (*M* − SD) was classified as the low group (see Figure 2). The results from the simple slope analysis indicated that perceived stress strongly predicted depression in the low emotional intelligence group (*β* = 0.34, *t* = 8.42, *p* < 0.001). Similarly, perceived stress significantly predicted depression in the high emotional intelligence group (*β* = 0.25, *t* = 6.53, *p* < 0.001). However, the impact of perceived stress on depression was more pronounced for individuals with low emotional intelligence compared to those with high emotional intelligence. Specifically, as perceived stress increased, depression increased to a greater extent for individuals with low emotional intelligence compared to those with high emotional intelligence.

**Table 3:** Coefficients for the tested moderated mediation model.

Predictors	Model 1 (rumination)			Model 2 (depression)		
	*β*	*SE*	*t*	*β*	*SE*	*t*
Gender	−0.10	0.04	−2.68	0.04	0.03	1.30
Age	−0.01	0.01	−0.31	−0.01	0.01	−0.12
Year of class	0.05	0.25	−0.30	0.73*	0.31	2.34
Perceived stress	0.45	0.03	14.13	0.30***	0.04	9.60
Rumination				0.36**	0.04	10.55
Emotional intelligence				−0.41*	0.03	−12.14
Perceived stress × emotional intelligence				−0.11*	0.05	1.97
*R* ^2^	0.26			0.61		
*F*	51.18***			122.47***		

× Represents the interaction item of perceived stress × emotional intelligence. *p < 0.05, **p < 0.01, ***p < 0.001.

## 4 Discussion

Grounded in the perseverative cognition hypothesis and Diathesis-stress models, the study constructed a moderated mediating model to explore the inner mechanisms of the relationship between perceived stress and depression in medical students. The result found that rumination served as a mediator in the relationship between perceived stress and depression. Furthermore, emotional intelligence moderated the link between perceived stress and depression. This is the first study to investigate the relationship between perceived stress and depressive in medical students through the effects of both cognitive and personality factors, offering valuable insights for the prevention and treatment of depression among medical students.

The results revealed that senior medical students exhibited higher levels of depression and rumination. It may be attributable to the progressively greater challenges and pressures faced by senior students, including increased academic workload, clinical rotation demands, and career preparation stress, which may contribute to elevated depressive symptoms. The result also found that rumination mediated the relationship between perceived stress and depression, which is consistent with previous research. A longitudinal study of U. S. adolescents demonstrated that rumination mediated the longitudinal association between stressful life events and anxiety symptoms across both adolescents and adults, regardless of gender (Polanco-Roman et al., 2016). Building upon this foundation, the current study further reveals that rumination also serves as a mediator in the relationship between perceived stress and depression. Characterized by repetitive contemplation of negative events and associated feelings, rumination represents a maladaptive form of cognitive self-reflection (Hu et al., 2014; Chen et al., 2023). This sustained engagement in negative emotion and cognition has been established to contribute to various detrimental health outcomes, including depression and anxiety (Watkins and Roberts, 2020). The findings indicate that perceived stress functions as a distal causal factor, while rumination acts as a proximal cause of depression. The distal factor (perceived stress) influences depression through proximal factors (rumination). This is consistent with previous research indicating that distal factors (e.g., social anxiety) influence depression via intermediate factors (e.g., rumination) (Kong et al., 2020). Our findings contribute to the advancement of existing theoretical frameworks based on earlier studies.

After controlling for gender, age, and year of class, the study found a positive correlation between year of class and depression among medical students. This relationship may stem from senior students’ exposure to compounding stressors (e.g., clinical rotations, licensing exams) and their amplified stress perception, both of which are known risk factors for depression. The results also indicate that emotional intelligence plays a moderating role in the direct path of this mediating process, which is consistent with previous findings. Specifically, a study on depressed adolescents in Iran, the Middle East, found that emotional intelligence could mitigate the relationship between perceived stress and suicidal ideation among adolescents hospitalized for depression (Abdollahi et al., 2016). Medical students face significant stressors, such as academic demands, frequent exams, an extended curriculum, and future-related anxieties (Wang et al., 2021; Gazzaz et al., 2018). However, elevated stress levels do not uniformly result in depression in all medical students. In comparison among medical students with high emotional intelligence, those with lower emotional intelligence experience a faster increase in depressive symptoms as their perceived stress intensifies. High levels of emotional intelligence effectively serve to mitigate the effect of perceived stress on depression. The finding indicates that medical students with stronger emotional regulation abilities can significantly reduce the risk of experiencing depressive feelings resulting from perceived stress. Emotional intelligence among medical students, acting as a positive psychological resource, constitutes a key safeguard. It can be selectively enhanced through targeted intervention measures to alleviate emotional adaptation issues arising from heightened perceived stress.

### 4.1 Implications

This study has revealed the underlying mechanisms of perceived stress in the development of depression among medical students, offering both theoretical significance and practical value. This research not only enhances our understanding of how perceived stress directly and indirectly affects depression in medical students, but also sheds light on the differences in the strength of the mediating effects among individuals with varying levels of emotional intelligence. From a practical standpoint, exploring the mechanisms behind depression among medical students has important implications for its prevention and intervention. The study identifies medical students who are at risk of depression, particularly those with high Perceived stress and low emotional intelligence. To lower depression risk stemming from medical school pressures (academic workload, social interactions, and clinical training), interventions should focus on modifying cognitive appraisal processes. By reframing stress interpretation, students may develop adaptive coping responses that reduce subjective stress burden. Additionally, it is essential to reduce repetitive rumination of negative emotions while enhancing their emotional awareness and regulation skills. This approach effectively reduces the risk of depression and promotes psychological well-being. Therefore, educators in medical institutions should conduct training programs aimed at improving emotional intelligence, thus lowering the effects of perceived stress on depression, thereby decreasing the prevalence of depression among medical students. For example, medical schools should consider implementing Emotion Management courses to teach psychological principles of emotion recognition, expression, and regulation. These courses could incorporate role-playing exercises and group counseling sessions to train emotional awareness skills. Additionally, educators may design realistic simulation scenarios—such as campus interpersonal conflicts and academic stress—using case study analysis and psychodrama techniques. This approach would help students internalize emotion regulation strategies in real-life learning contexts, leading to measurable improvements in emotional intelligence.

### 4.2 Limitation and future directions

The research has various constraints that need to be considered in subsequent studies. Firstly, the cross-sectional design restricts the capacity to determine causal relationships among the variables, longitudinal or experimental designs need to be used in the future study. Secondly, the sample selection from a single university in a specific city necessitates caution when extending these findings to broader populations. Thirdly, it is noteworthy that rumination can be differentiated into two forms: reflective rumination and brooding rumination. Prior studies have demonstrated that these two manifestations of rumination exhibit diverse psychological effects on individuals (Whitmer and Gotlib, 2011). Future investigations could explore the distinct impacts of these rumination types on depressive affect in medical students. Additionally, considering the demonstrated efficacy of high emotional intelligence in attenuating depressive emotions and mitigating the adverse effects of perceived stress, there is potential value in developing targeted programs for enhancing emotional intelligence to mitigate the risk in medical students’ depression.

## 5 Conclusion

In conclusion, this study represents a pioneering effort in exploring how rumination mediates and emotional intelligence moderates the correlation between perceived stress and depression among medical students. It provides insight into the intricate relationship between how, when, and in what ways perceived stress is linked to depression. The findings contribute to the existing literature by identifying intervening and influencing factors within the connection involving perceived stress and depression. Specifically, rumination is identified as a potential mediating factor connecting perceived stress and depression. Additionally, emotional intelligence moderates this mediation, suggesting that higher emotional intelligence buffers the harmful effects of perceived stress on depression.

## Data Availability

The original contributions presented in the study are included in the article/supplementary material, further inquiries can be directed to the corresponding author.
